# Peer Review of "Postacute Sequelae of COVID-19 and Adverse Psychiatric Outcomes: Protocol for an Etiology and Risk Systematic Review"

**DOI:** 10.2196/45306

**Published:** 2023-03-14

**Authors:** Yin Qianlan

**Affiliations:** 1 Navy Medical University Shanghai China

**Keywords:** COVID-19, long COVID, post–COVID-19 condition, postacute sequelae of COVID-19, PASC, psychiatry, epidemiology, evidence-based medicine

*This is a peer-review report submitted for the paper “Postacute Sequelae of COVID-19 and Adverse Psychiatric Outcomes: Protocol for an Etiology and Risk Systematic Review.”*

## Round 1 Review

### Serious Concerns

*Do you have any serious concerns about the manuscript such as fraud, plagiarism, unethical or unsafe practices?*

No.

*Have authors’ provided the necessary ethics approval (from authors’ institution or an ethics committee)?*

Yes.

### Language Quality

*How would you rate the English language quality?*

High quality.

### Validity and Reproducibility

*Is the reasons for conducting the study and its objectives clearly explained?*

No.

*Is the study design appropriate?*

Yes.

*Are sufficient details provided so that the method can be replicated?*

Yes.

*Are datasets available so that others could use them?*

Not applicable.

### Suggestions

*Based on your answers in section 3 how could the author improve the protocol?*

As an important part of a review is the declaration of the purpose of the protocol [1], the Introduction section should be the core of the article. However, after reading the beginning of the paper, I realized the seriousness of COVID-19, but I could not see the key point of the research. There are a lot of data to emphasize the worse outcomes, but I do not know how these data contributed to the relationship between the major topic of postacute sequelae of COVID-19 and adverse psychiatric outcomes; for example, the narrative on the effect of therapies. Hence, a more organized structure for the Introduction section with more conciseness would be easier for the readers.

### Decision

*Requires revisions:* The manuscript contains objective errors or fundamental flaws that must be addressed and major revisions are suggested.
